# Addicted to AI? Prevalence and psychological drivers of generative AI dependency among pre-service teachers

**DOI:** 10.3389/fpsyg.2026.1922716

**Published:** 2026-08-12

**Authors:** Bin Jiang, Zhuo Wang

**Affiliations:** School of Educational Science, Qingdao University, Qingdao, China

**Keywords:** generative AI, AI dependency, pre-service teachers, impulsivity, anthropomorphic attachment, perceived AI literacy

## Abstract

**Introduction:**

Generative artificial intelligence (GenAI) has become embedded in higher education, raising concerns about students’ behavioral dependency on these tools. Focusing on pre-service teachers—who will shape how a future generation learns to use AI—we asked how prevalent GenAI dependency is, what drives it, and whether perceived AI literacy protects against it.

**Methods:**

In a mixed-methods design, 565 education-major students in Chinese teacher-preparation programs completed an online survey, and five were interviewed.

**Results:**

Dependency markers were already widespread: 79% called AI indispensable to their learning, 78% felt unaccustomed without it, 54% “asked AI first,” and 31% used its answers without verification. In the structural model (*R*^2^ = 0.60), the strongest drivers were *impulsivity* (*β* = 0.40) and *anthropomorphic attachment* (*β* = 0.28), with a smaller contribution from AI anxiety (*β* = 0.15); trust was non-significant. Necessary condition analysis identified social norms as a necessary-in-degree condition. Perceived AI literacy was modestly *protective* (*β* = −0.19), stronger in the full model than its zero-order correlation (*r* = −0.14)—a suppression effect, because more AI-literate students also trusted AI more and sat within more AI-reliant peer groups. Interviews converged with the survey model, most strikingly on anthropomorphic attachment, while qualifying the smaller anxiety pathway.

**Discussion:**

Dependency is driven chiefly by dispositional impulsivity and affective attachment rather than by low competence; interventions—for policymakers, teacher educators, and pre-service teachers—should prioritize self-regulation and awareness of parasocial engagement, treating AI-literacy training as a secondary lever.

## 1 Introduction

The rapid diffusion of generative artificial intelligence (GenAI) tools—such as ChatGPT, DeepSeek, and Doubao—has transformed how university students search for information, draft assignments, and prepare lessons. Within barely two years of ChatGPT’s public release, GenAI use among university students moved from novelty to near-ubiquity, and reviews now document its penetration across virtually every discipline and stage of the study cycle (Panda, 2025). For many students the tools have become a default first step rather than an occasional aid, shifting scholarly attention from whether students use GenAI to how their use is structured and what it displaces.

Alongside documented benefits, educators increasingly worry that habitual use is shading into dependency: a pattern in which students reflexively delegate cognitive work to AI, struggle to complete tasks without it, and accept its outputs without scrutiny. Experimental work shows that large language models can reduce the mental effort students invest in inquiry while compromising the depth of their reasoning (Stadler et al., 2024), and survey research links heavier reliance on AI to weaker engagement in critical thinking through cognitive offloading (Gerlich, 2025). Recent work has begun to treat over-engagement with GenAI as a form of dependence or even addiction in its own right (Du et al., 2026; Zahid et al., 2026).

For pre-service teachers, this risk is doubled. As current learners, they are exposed to the same offloading dynamics as their peers; as future educators, the habits and judgment they form now will shape how a next generation of pupils learns to use—or over-rely on—AI. A teacher who cannot work without AI, or who accepts its outputs uncritically, is poorly placed to cultivate independent thinking in others.

A first generation of empirical studies has begun to map the antecedents of AI dependency (Zhang et al., 2024; Li and Xing, 2025), and in the teacher context. Du et al. (2026) applied the I-PACE model and found cognitive absorption to be the strongest predictor of teachers’ GenAI addiction. What remains underexamined is (a) how prevalent behavioral dependency already is among pre-service teachers, (b) which antecedents drive it once their shared variance is accounted for, and (c) whether the competence most often proposed as protective—AI literacy—actually buffers against it. We address these questions with a multi-method design: we estimate prevalence, use SEM to identify the net drivers of dependency, use NCA to test necessity, and use fsQCA as an exploratory configurational probe. Specifically, we were aiming to address the following:

*RQ1:* How prevalent are behavioral GenAI dependency and its markers among pre-service teachers, and how do they perceive its risks?

*RQ2:* Which psychological and social antecedents drive dependency?

*RQ3:* Is any antecedent a necessary condition?

*RQ4:* Do the antecedents combine into distinct sufficient configurations?

## 2 Theoretical background and hypotheses

We conceptualize GenAI dependency specifically as behavioral over-reliance: a tendency to default to AI for tasks one could perform independently, a functional difficulty in completing academic work without it, and the uncritical adoption of its outputs without verification. We deliberately bound the construct to observable behavior rather than to the affective experience of dependence; although such affective reactions are related—and are central to addiction conceptualizations such as the Generative AI Addiction construct of Du et al. (2026), whose items include withdrawal-like unease—they belong to a distinct emotional facet. This behavioral framing also distinguishes dependency from mere frequent or skilled use and from momentary automation bias, locating it in the cognitive-offloading account of problematic technology use (Risko and Gilbert, 2016), in which delegating effortful processing gradually displaces self-regulated cognition (Zimmerman, 1990; Bjork et al., 2013).

Drawing on three complementary literatures—problematic technology use, technology acceptance, and human–AI relationships—we advance a directional hypothesis for each of six antecedents.

### 2.1 Impulsivity

A preference for immediate reward over effortful, delayed payoff is a classic driver of compulsive technology use (Patton et al., 1995).

*H1:* Impulsivity is positively associated with dependency.

### 2.2 AI anxiety/fear of missing out

Fear of missing out has been robustly linked to compulsive digital engagement (Przybylski et al., 2013; Elhai et al., 2016, 2020).

*H2:* AI anxiety/FOMO is positively associated with dependency.

### 2.3 Trust in AI

Research on trust in automation identifies a performance- or reliability-based dimension—the belief that a system is competent and its outputs accurate—as the principal cognitive driver of reliance on that system (Lee and See, 2004; McKnight et al., 2011). When such trust is miscalibrated relative to actual reliability, users over-rely: they accept outputs uncritically and forgo verification, the pattern documented as automation bias (Parasuraman and Riley, 1997; Buçinca et al., 2021). Consistent with this literature and with technology-acceptance accounts of perceived usefulness (Davis, 1989), our two trust items were written to capture this accuracy/reliability belief about GenAI.

*H3:* Trust in AI is positively associated with dependency.

### 2.4 Anthropomorphic attachment

When a student experiences AI as a quasi-social partner, reliance may extend into the affective domain (Epley et al., 2007; Skjuve et al., 2021).

*H4:* Anthropomorphic attachment is positively associated with dependency.

### 2.5 Perceived AI literacy

AI literacy—the competencies that enable people to use, evaluate, and critically interrogate AI (Long and Magerko, 2020; Ng et al., 2025)—is theorized as protective (Gerlich, 2025). We measured it, as is standard, through self-report; the construct is therefore more precisely *perceived* AI literacy—students’ confidence in their own competence—rather than tested proficiency, a distinction we return to in the Discussion.

*H5:* Perceived AI literacy is negatively associated with dependency.

### 2.6 Social norms

When AI use is normalized within a peer group, even reserved students face a normative pull toward reliance (Venkatesh et al., 2003).

*H6:* Social norms are positively associated with dependency.

## 3 Method

### 3.1 Participants and procedure

Data were collected via an online questionnaire distributed through WeChat, using an anonymous convenience and peer-referral (snowball) sampling strategy, from university students enrolled in education-related majors within teacher-preparation tracks. Because recruitment was anonymous and propagated by peer forwarding, we could neither control nor independently verify the regional or year-of-study composition of the realized sample; these features are best regarded as uncontrolled characteristics of a convenience sample rather than a designed sampling frame. Respondents’ IP addresses resolved to 12 provincial-level regions but were predominantly in Shandong (96.1%), concentrated in Jinan (50.4%) and Qingdao (37.0%); institutional affiliation was not collected and cannot be recovered from IP data. Participation was voluntary, and informed consent was obtained online before any item could be accessed. From 596 responses collected, we removed 31 (5.2%): 29 who reported never having used GenAI—20 of whom also completed the survey in under 60 s—and 2 further respondents who completed in under 60 s, yielding a clean analytic sample of *N* = 565 in which no respondent was a non-user or sub-60-s completer. It was predominantly female (76.8%, *n* = 434) and, by year of study, comprised first-year (60.5%), second-year (3.7%), third-year (28.7%), and fourth-year (7.1%) students.

### 3.2 Measures

All constructs were measured on 7-point Likert scales (1 = strongly disagree, 7 = strongly agree). Because behavioral GenAI dependency and several antecedents are emerging constructs for which no validated Chinese instrument for this population existed at the time of design, all scales were developed for this study following suggestions of Du (2024), such as item development informed by established conceptualizations, expert content review, psychometric validation, and cross-validation on an independent sample. Full item wording and standardized loadings appear in Appendix A. The outcome, dependency (DEP), comprised four behavioral items. Six antecedents were measured: impulsivity (IMP), AI anxiety/FOMO (ANX), trust (TRU), anthropomorphic attachment (ANTH), perceived AI literacy (LIT), and social norms (NORM).

Initially, all items were reviewed for content validity and question clarity by three educational-research experts (each with 13 + years of experience) and pretested on an initial development sample, in which the problematic items below were identified. Specifically, three items were removed during scale development, using the initial development sample for item screening. First, a reverse-worded impulsivity item was not processed as intended: it correlated positively, not negatively, with the other two impulsivity items (*r* = 0.32 and 0.39) and with dependency (*r* = 0.28), and reverse-keying it collapsed the subscale’s alpha to a negative value (−0.11); it was dropped rather than recoded. Second, a social-norms item cross-loaded onto the dependency dimension, correlating more strongly with dependency (*r* = 0.51) than with the retained norms items (*r* = 0.24), and was removed. Third, a further social-norms item tapping tool availability (“AI is always available, with almost no barrier”), conceptually distinct from the two descriptive-norm items about peer use, loaded weakly (standardized loading = 0.50) and was removed, leaving a two-item descriptive-norms scale with a strong Spearman–Brown coefficient (0.89).

All decisions were made on the development sample before the confirmatory analysis; the refined scales then replicated in the independent sample (impulsivity Spearman–Brown = 0.76; social norms Spearman–Brown = 0.88). Moreover, reliability and convergent validity were adequate for all constructs (Cronbach’s *α* or, for two-item scales, the Spearman–Brown coefficient recommended by Eisinga et al. (2013), ≥ 0.69—the two-item trust scale being marginally below the 0.70 convention at 0.69; CR ≥ 0.69; AVE ≥ 0.53). Two constructs (trust and descriptive norms) were measured with two items each; such two-indicator factors are statistically identified when embedded in a larger multi-factor model, as they are here, in line with the two-indicator rule for structural equation models (Bentler and Chou, 1987; Kline, 2016). In latent-variable SEM, convergent validity is judged primarily through composite reliability and average variance extracted (AVE) rather than through coefficient *α* (Fornell and Larcker, 1981; Bagozzi and Yi, 1988). By these criteria the two-item trust scale was adequate (CR = 0.69 ≥ 0.60; AVE = 0.53 > 0.50), and its *α*/Spearman–Brown value marginally below the 0.70 convention is partly a mechanical consequence of scale length, because coefficient *α* is deflated for short scales even when the average inter-item correlation is respectable (Cortina, 1993); we nonetheless retain and flag it transparently rather than trim a theoretically specified construct. A confirmatory factor analysis (CFA) of the seven-factor model fit well (robust CFI = 0.927, TLI = 0.909, RMSEA = 0.061, SRMR = 0.045). Discriminant validity was supported by the Fornell–Larcker criterion and HTMT ratios below 0.85. Reliability and validity are reported in section 4.2.

### 3.3 Analytic strategy

We adopted a deliberately multi-method analytic strategy because the antecedents of behavioral dependency can relate to the outcome in three logically distinct ways—as net contributors, as necessary preconditions, and as components of sufficient configurations—that no single technique captures. Regression-based methods, including SEM, estimate the average, additive, and symmetric net effect of each predictor holding the others constant; they answer which factors, on balance, raise or lower dependency. They cannot establish whether a factor is a necessary bottleneck without which dependency cannot occur, nor whether factors combine into distinct sufficient “recipes” (Dul, 2016; Woodside, 2013). Because net effect, necessity, and sufficiency are not reducible to one another (Vis and Dul, 2018), we triangulated three complementary techniques, each matched to one question, and treated their convergence or divergence as itself informative.

Descriptive statistics addressed the prevalence question (RQ1). The sample (*N* = 565) is well above the conventional minimum of about 200 cases for SEM (Kline, 2016). A Monte Carlo power analysis (1,000 replications) confirmed that the study was well powered to detect the substantive paths—power ≈ 1.00 for impulsivity, attachment, and perceived literacy, and 0.89 for anxiety—although it was underpowered for the small, non-significant trust path (0.37); all power values are reported in Table 1.

To identify the net drivers of dependency (RQ2), we used latent-variable structural equation modeling. SEM was appropriate for three reasons. First, our constructs are latent and each is measured by multiple self-report items; SEM models them as latent factors and partitions item variance into true-score and measurement-error components, so the structural estimates are disattenuated for unreliability rather than assuming, as composite-score regression does, that each scale is measured without error (Bollen, 1989; Kline, 2016). Second, the six antecedents are moderately intercorrelated; SEM estimates each one’s unique contribution with the others controlled, isolating net effects and making suppression detectable. Third, SEM yields a global test of how well the hypothesized structure reproduces the observed covariances (Anderson and Gerbing, 1988). We estimated the model in lavaan (Rosseel, 2012) with a robust maximum-likelihood estimator (MLR), whose standard errors and fit statistics are robust to the non-normality typical of Likert data, and corroborated the latent model with a composite-score regression.

To test whether any antecedent is a necessary condition (RQ3), we conducted Necessary Condition Analysis (NCA; Dul, 2016). NCA answers a question regression and SEM cannot. Additive models estimate how much the average level of the outcome shifts with a predictor and allow a high value on one factor to compensate for a low value on another; a necessary condition, by contrast, must be in place for the outcome to occur at all, and its absence cannot be offset by any other factor. Necessity is thus a claim about a ceiling—the maximum outcome attainable at a given level of the condition—not about an average slope, and a condition can be necessary while having a small or even null net effect (Dul, 2016; Vis and Dul, 2018). NCA fits a ceiling line over the empty (upper-left) region of the condition–outcome plot and expresses necessity as an effect size *d*, with significance obtained by permutation (Dul et al., 2020). Testing necessity separately guards against dismissing an enabling precondition merely because its net regression effect is modest.

Finally, as an exploratory step we examined whether the antecedents combine into distinct sufficient configurations (RQ4), using fuzzy-set Qualitative Comparative Analysis (fsQCA; Ragin, 2008). fsQCA is a set-theoretic, case-oriented method resting on assumptions opposite to those of regression: rather than isolating each variable’s net effect, it asks how conditions operate in combination (conjunctural causation), permits several different combinations to reach the same outcome (equifinality), and treats the presence and absence of the outcome asymmetrically (Fiss, 2011; Woodside, 2013). Conditions and outcome are calibrated into fuzzy-set membership scores, and truth-table minimization identifies configurations consistently sufficient for high—and, separately, low—dependency, evaluated by consistency and coverage (Greckhamer et al., 2018; Pappas and Woodside, 2021; Schneider and Wagemann, 2012). Configurational analysis is most informative when conditions vary substantially across cases; because several of ours were strongly skewed (perceived literacy and social norms were near-universal), we treated fsQCA as an exploratory robustness check rather than a primary basis for inference (Section 4.5).

**Table 1:** Standardized SEM paths predicting dependency (*N* = 565).

Predictor	*β*	*p*	zero-order *r*	MC power
Impulsivity	0.40	<0.001 ***	0.58	1.00
AI anxiety/FOMO	0.15	0.005 **	0.49	0.89
Trust in AI	0.11	0.187 ns	0.41	0.37
Anthropomorphic attachment	0.28	<0.001 ***	0.45	1.00
Perceived AI literacy	−0.19	<0.001 ***	−0.14	1.00
Social norms	0.08	0.143 ns	0.13	0.42
*R*^2^ (DEP)	0.60			

**p < 0.01; ***p < 0.001.

### 3.4 Qualitative strand

To interpret and triangulate the survey findings, we adopted a sequential explanatory mixed-methods design (quan → QUAL). From survey respondents who consented to follow-up, we purposively sampled five informants varied by gender and year of study (three women, two men; two first-year, two third-year, one fourth-year, spanning primary education, early-childhood, psychology, and instructional technology tracks). Semi-structured telephone/online interviews (22–56 min) were audio-recorded, transcribed verbatim, and analysed with deductive thematic analysis (Braun and Clarke, 2006) using a coding manual derived from the six antecedent constructs plus two emergent themes (guilt/ambivalence about reliance; teacher irreplaceability). Thirty-eight segments were coded independently by two coders; inter-coder agreement was strong (Cohen’s *κ* = 0.88; 89% raw agreement, 34/38), and the four disagreements—all at construct boundaries (attachment vs. trust, anxiety vs. literacy, literacy vs. critical verification)—were resolved by consensus. The interview guide is provided in Appendix B.

## 4 Results

### 4.1 Prevalence of dependency markers (RQ1)

Markers of behavioral dependency were already widespread among these pre-service teachers (Table 2). A majority reported that AI had become an indispensable part of their learning (79% agreed; *M* = 5.26), that they felt unaccustomed after a period without it (78%), and that their first reaction to a task was to “ask AI first” (54%). Nearly half habitually delegated to AI even tasks they could complete themselves (44%), and about a third routinely adopted its answers without deeper thought (34%) or without bothering to verify them (31%). Trust was correspondingly high: 71% believed AI’s answers were generally accurate. At the same time, a substantial minority voiced professional unease: 44% worried that long-term reliance would erode their teaching ability, and 47% wished to reduce their dependence. Dependency is thus a prevalent pattern that coexists with the students’ own awareness of its risks.

**Table 2:** Prevalence of GenAI dependency markers (*N* = 565).

Item (abbreviated)	*M*	% agree (≥5)
AI has become an indispensable part of my learning	5.26	79
After a while without AI, I feel unaccustomed	5.33	78
I believe AI’s answers are generally accurate (trust)	4.99	71
My first reaction to a task is “ask AI first”	4.51	54
Even doable tasks, I hand to AI first	4.10	44
I adopt AI answers without thinking deeply	3.74	34
I cannot be bothered to verify AI’s conclusions	3.55	31
I worry reliance will erode my teaching ability	4.81	44
I hope to reduce my AI dependence	5.06	47

### 4.2 Measurement model and independent validation

The seven-factor CFA fit the primary data well (CFI = 0.927, TLI = 0.909, RMSEA = 0.061, SRMR = 0.045), with all standardized loadings significant. Reliability and validity met conventional thresholds (Table 3), the only marginal case being the two-item trust scale (Spearman–Brown = 0.69). The measurement model held in the independent sample (*N* = 144): the CFA fit acceptably (CFI = 0.857, RMSEA = 0.083) and reliabilities were comparable (including trust, Spearman–Brown = 0.77). Critically for the concern that item removal might be *post hoc*, the two item-reduced scales retained strong reliability in this independent sample (impulsivity Spearman–Brown = 0.76; social norms Spearman–Brown = 0.88), indicating that the revised measurement structure is stable across fresh data rather than an artifact of the development sample. Because all measures were self-reported, we tested for common method bias: Harman’s single-factor test yielded a first unrotated factor explaining only 33.1% of the variance (well below 50%), and a common-latent-factor model estimated shared method variance at roughly 16% while leaving the focal structural paths essentially unchanged (impulsivity *β* = 0.40 → 0.30; attachment *β* = 0.28 → 0.25), indicating that method bias does not account for the findings. The heterogeneous correlations in Table 4 (*r* = 0.58 for dependency with impulsivity but −0.14 with perceived literacy) are likewise inconsistent with a single pervasive method factor. Strong social-desirability suppression is also unlikely: the dependency mean sat mid-scale (3.89/7, not floored) and about a third of respondents openly endorsed undesirable behaviors such as not verifying AI output (31%) and adopting it without thinking (34%).

**Table 3:** Constructs, reliability, and validity (primary sample, *N* = 565).

Construct	Items	*M* (*SD*)	*α*/*SB*	*CR*	*AVE*
Impulsivity (IMP)	2	4.24 (1.44)	0.79 (SB)	0.79	0.66
AI anxiety/FOMO (ANX)	6	4.14 (1.43)	0.90	0.90	0.60
Trust in AI (TRU)	2	4.87 (1.04)	0.69 (SB)	0.69	0.53
Anthropomorphic attachment (ANTH)	2	3.96 (1.58)	0.79 (SB)	0.79	0.65
Perceived AI literacy (LIT)	3	5.76 (0.99)	0.86	0.86	0.67
Social norms (NORM)	2	5.87 (1.00)	0.89 (SB)	0.89	0.81
AI dependency (DEP)	4	3.89 (1.46)	0.91	0.91	0.71

**Table 4:** Means, SDs, and correlations (*N* = 565).

	IMP	ANX	TRU	ANTH	LIT	NORM	DEP
IMP	1						
ANX	0.52	1					
TRU	0.39	0.31	1				
ANTH	0.31	0.23	0.42	1			
LIT	−0.09	−0.04	0.19	0.08	1		
NORM	0.16	0.17	0.29	0.03	0.38	1	
DEP	0.58	0.49	0.41	0.45	−0.14	0.13	1

Correlations ≥ |0.09| are significant at *p* < 0.05. Perceived AI literacy (LIT) correlates positively with trust and norms, negatively with impulsivity, and negatively and weakly with dependency.

### 4.3 Drivers of dependency: SEM (RQ2)

**Figure 1:**
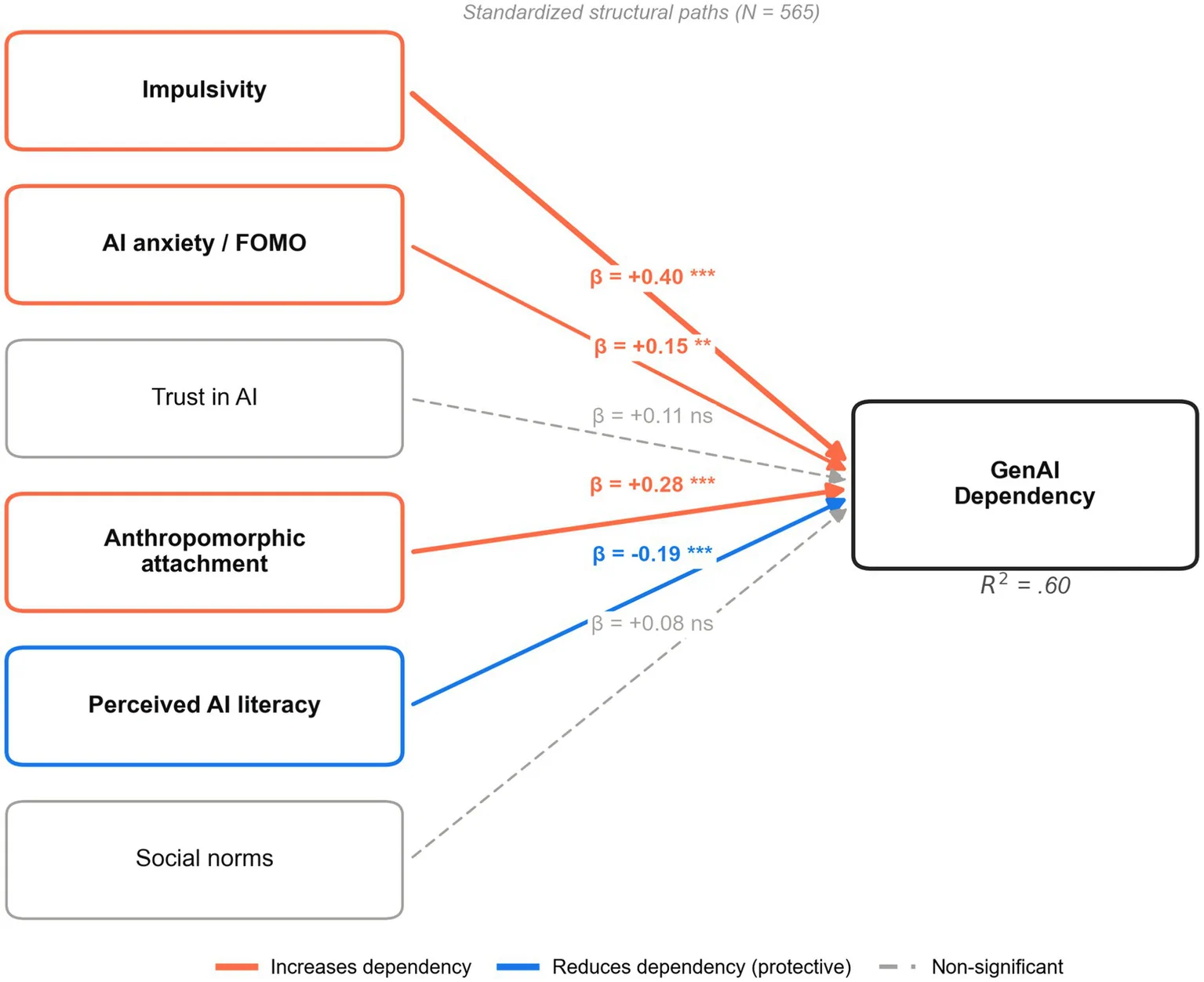
Structural model of GenAI dependency (standardized paths, *N* = 565).

The structural model fit well (CFI = 0.927, RMSEA = 0.061, SRMR = 0.045) and explained 60% of the variance in dependency (Table 1; Figure 1). The strongest drivers were impulsivity (*β* = 0.40, *p* < 0.001) and anthropomorphic attachment (*β* = 0.28, *p* < 0.001), with a smaller significant contribution from AI anxiety (*β* = 0.15, *p* = 0.005), supporting H1, H2, and H4. Trust was positive but non-significant (*β* = 0.11, *p* = 0.19), and social norms were unrelated (*β* = 0.08, ns), so H3 and H6 were not supported. Consistent with H5, perceived AI literacy had a significant *protective* effect (*β* = −0.19, *p* < 0.001). This protective role was partly muted at the bivariate level (zero-order *r* = −0.14) because perceived literacy correlated positively with trust (*r* = 0.19) and social norms (*r* = 0.38), both associated with greater dependency; partialling out these overlapping influences strengthened its protective contribution—a partial suppression pattern, which suggests that analyses relying on simple correlations may underestimate the protective role of perceived AI literacy. The Monte Carlo power analysis confirmed the substantive effects were adequately powered at *N* = 565 (Table 1), and the same pattern emerged in a composite-score regression.

### 4.4 Necessity: NCA (RQ3)

**Figure 2:**
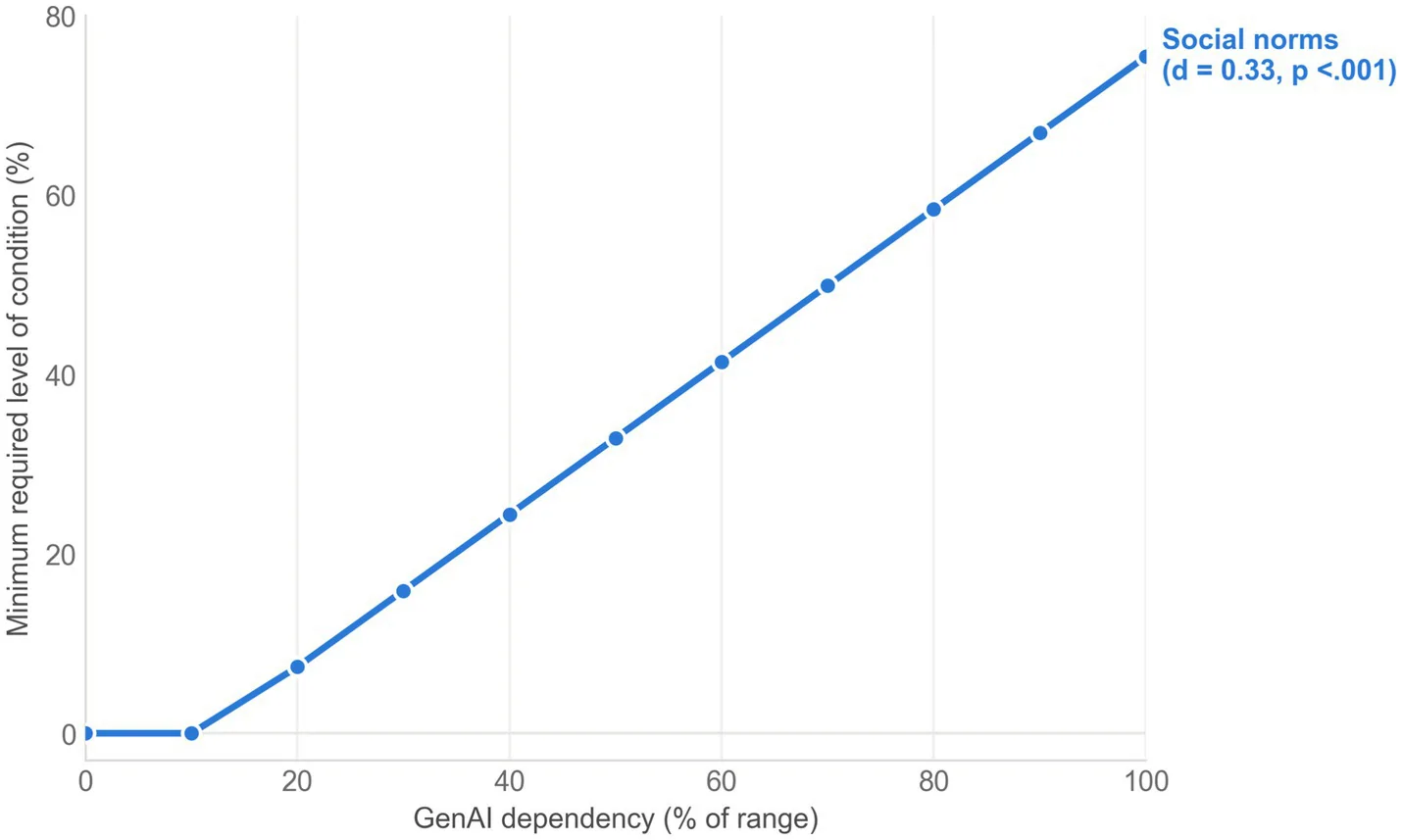
NCA bottleneck: social norms as the necessary condition (*N* = 565).

NCA identified social norms as necessary in degree (*d* = 0.33, *p* < 0.001, see Figure 2): an environment in which AI use is normative must be in place for strong dependency to develop, yet norms do not drive the *level* of dependency (non-significant in the SEM). No other condition reached necessity—trust (*d* = 0.08, *p* = 0.23), impulsivity and anxiety (*d* = 0.00), attachment (*d* = 0.01), and perceived literacy (*d* = 0.20, *p* = 0.07). SEM and NCA are thus complementary: impulsivity and attachment set the level of dependency, whereas social norms function as an enabling precondition.

### 4.5 Exploratory configurational analysis: fsQCA (RQ4)

As a final, exploratory step we examined whether a configurational lens would add anything beyond the variable-centered analyses, conducting fuzzy-set QCA (Ragin, 2008) on the calibrated conditions using theory-based anchors and standard frequency and consistency–PRI thresholds. Because several conditions are strongly skewed—perceived literacy and social norms are near-universal in this sample—the solutions were highly calibration-sensitive, and once the PRI threshold was enforced the analysis recovered no configuration beyond, or inconsistent with, the SEM and NCA results: high dependency corresponded to the joint presence of the same dispositional and affective drivers already identified, and no additional equifinal route emerged. Because fsQCA therefore added nothing new and its solutions were unstable under this skew, we do not report the truth tables in detail and base inference on the SEM and NCA. This convergence is nonetheless reassuring: the configurational analysis contradicts none of the variable-centered findings.

### 4.6 Qualitative triangulation (RQ2, RQ3)

The five interviews cohered into recognizable profiles that mapped onto the structural model (Table 5). The clearest convergence concerned *anthropomorphic attachment*: the informant who most vividly personified AI (“I want AI to be my classmate, my friend, my teacher”) also reported the most difficulty working without it, whereas the two who insisted AI is “just a tool… the hands and feet, while the person is the brain” were the least reliant—triangulating the SEM result that attachment, more than any attitude, predicts dependency more strongly. *Impulsivity* surfaced just as concretely (“writing it myself takes over an hour; with AI, five minutes”). A trust-versus-verification gradient distinguished the more reliant first-years, who judged AI “rarely wrong,” from the critical upper-years, who caught fabricated citations and deemed only 20–30% of AI output usable. Two honest qualifications emerged. First, AI anxiety/FOMO was real but not universal—only one informant described peer-comparison anxiety (“a classmate’s AI work rivals a master’s student’s… I feel the gap”), while three explicitly denied any peer pressure—consistent with anxiety’s smaller net effect in the SEM. Second, perceived literacy did not translate cleanly into protection: one first-year articulated full agency (“whatever I can write myself, I write, or my mind atrophies”) yet offloaded heavily and felt guilt, illustrating the gap between perceived competence and protective behavior that helps explain perceived literacy’s modest, suppression-masked effect. A theme common to all five—teacher irreplaceability and “human care AI cannot give”—echoed the professional unease evident in the prevalence data.

**Table 5:** Qualitative triangulation of the survey findings (five informants; Cohen’s κ = 0.88).

Survey finding	Illustrative interview evidence	Convergence
Impulsivity → higher dependency (*β* = 0.40)	“Writing it myself takes over an hour; with AI, five minutes” (M, Yr 1); delegates even self-criticism essays	Converges
Anthropomorphic attachment → higher dependency (*β* = 0.28)	“I want AI to be my classmate, my friend, my teacher” (F, Yr 1); the two who called AI “just a tool/hands and feet” were least reliant	Converges (strongest)
Trust enables reliance; critical verification protects	High-trust: “AI rarely errs, mostly correct” (F, Yr 1); critical informant catches fabricated citation numbers, deems 20–30% usable (F, Yr 4)	Converges
AI anxiety/FOMO — a smaller predictor (*β* = 0.15)	“A classmate’s AI work rivals a master’s student’s… I feel the gap” (F, Yr 3); yet three of five denied any peer pressure	Partial — real but not universal
Perceived literacy — modest, suppression-masked protection	Agentic senior: “steer AI with my own thinking; over-trusting it and losing the ability to think is frightening”; yet a first-year voiced the same agency while offloading heavily	Converges with suppression reading
Norms as a necessary floor (NCA)	“Everyone around me uses it — it’s just an everyday tool” (F, Yr 1); “peer pressure pushes you to learn AI” (F, Yr 3)	Converges
High prevalence + professional unease (RQ1)	All five used AI routinely for study and lesson-prep; all invoked teacher irreplaceability (“human care AI cannot give”)	Converges

## 5 Discussion

This study asked how prevalent behavioral GenAI dependency is among pre-service teachers, what predicts it, and whether perceived AI literacy protects against it.

First, behavioral GenAI dependency is already widespread. Four in five of these future teachers who are currently using GenAI regard AI as indispensable to their learning, a majority default to consulting it first, and about a third accept its outputs without verification. That such patterns are widespread in a population who will soon model learning habits for pupils lends urgency to the concern that habitual offloading erodes self-regulated cognition (Zimmerman, 1990; Bjork et al., 2013). Notably, these students are not naive about the risk: nearly half worry that reliance will erode their teaching ability, a self-awareness that intervention efforts can build upon.

Second, dependency is most strongly associated with dispositional impulsivity and affective attachment. Impulsivity and anthropomorphic attachment were the strongest predictors, with anxiety making a smaller contribution and trust non-significant. This converges with Du et al. (2026), who found teachers’ GenAI addiction driven most strongly by cognitive absorption—an immersive form of engagement—rather than by instrumental beliefs such as perceived usefulness: across both studies, the strongest routes into over-reliance run through immersive and dispositional processes rather than reasoned, instrumental appraisal. The prominence of anthropomorphic attachment is notable, suggesting that as conversational agents become more human-like, the parasocial bond students form with them becomes a genuine engine of dependency (Skjuve et al., 2021).

Third, perceived AI literacy is protective, but only modestly, and in a way masked at the surface by its positive overlap with trust and social norms. In the multivariate model perceived literacy reduced dependency (*β* = −0.19), yet its zero-order correlation was weaker (−0.14)—a partial suppression effect arising because students who perceive themselves as more AI-literate also tend to trust AI more and to be more embedded in AI-using peer norms, both of which raise dependency. Perceived literacy’s protective contribution thus emerges only once these countervailing associations are controlled. This nuance reconciles a mixed literature: studies that examine simple correlations may conclude that self-reported AI literacy is unrelated to dependency, whereas a model that separates perceived literacy from its correlates reveals a genuine, if small, buffering role. Two cautions temper the finding. Substantively, the effect is modest relative to the dispositional drivers, so perceived literacy is a secondary rather than a primary lever. Methodologically, our measure captured *perceived* rather than tested literacy, and near-universally high self-ratings may understate true variation; objective literacy tasks are needed to confirm it. Social norms, meanwhile, functioned as a necessary precondition (NCA) without driving the level of dependency.

## 6 Implications

### 6.1 For researchers

These findings bear on how the antecedents of AI dependency are theorized and measured. The dominance of impulsivity and anthropomorphic attachment over instrumental beliefs such as trust echoes Du et al. (2026)—whose immersive cognitive-absorption driver likewise outweighed instrumental beliefs—and suggests that dependency is better understood as a dispositional and experiential process than as a reasoned technology-adoption outcome; models built mainly on perceived usefulness or ease of use (Davis, 1989) may under-specify it, and parasocial-relationship constructs (Skjuve et al., 2021) warrant a more central place. The suppression pattern we observed—perceived literacy appearing unrelated to dependency at the bivariate level yet protective once its positive overlap with trust and norms is controlled—is also a methodological caution: designs relying on zero-order correlations may misjudge perceived literacy’s role, so multivariate models are needed to recover it. Finally, our near-ceiling, self-reported literacy measure points to a concrete measurement agenda: pairing self-report with performance-based (tested) literacy tasks and with behavioral traces of reliance would sharpen future estimates.

### 6.2 For teacher educators

The results redirect intervention away from AI-literacy instruction alone and toward the dispositional and affective roots of over-reliance. Because impulsivity was the strongest driver, the highest-yield strategy is likely to embed metacognitive self-regulation—goal setting, progress monitoring, and the deliberate tolerance of “desirable difficulties”—into coursework (Zimmerman, 1990; Bjork et al., 2013), so that pausing to think before delegating to AI becomes a trained habit. Because anthropomorphic attachment also drove reliance, programs might add brief reflective activities that make the parasocial pull of human-like agents visible—for instance, prompting students to notice when they treat a chatbot as a companion rather than a tool (Skjuve et al., 2021). Perceived AI literacy should still be taught, but as a complement rather than a stand-alone safeguard: because more self-assured students also trust AI more and are more embedded in AI-using peer norms, literacy training delivered without self-regulation support risks raising confident use without curbing dependency. Crucially, none of this warrants restricting access, which forgoes the documented benefits of well-managed AI use (Yang and Appleget, 2024); the aim is calibrated, self-regulated use rather than abstinence.

### 6.3 For pre-service teachers

The prevalence findings carry both a warning and an opening. The warning is that dependency markers are already near-ceiling in this population, and that the students most confident in their AI skills are not thereby protected—self-assessed competence can coexist with, and even accompany, heavy reliance. The opening is the self-awareness the data reveal: nearly half of respondents already wished to reduce their dependence and worried about its effect on their future teaching. This readiness is a resource that concrete, low-effort practices can build on—setting explicit limits on when to consult AI, attempting tasks unaided before turning to it, and routinely verifying its output rather than accepting it wholesale (a check a third of the sample admitted skipping). Because these future teachers will model AI habits for their own pupils, cultivating deliberate, self-regulated use now is not merely a personal skill but a professional responsibility.

## 7 Limitations and future research

Several limitations qualify these conclusions. The design was cross-sectional and cannot establish temporal ordering or rule out reverse causality; the SEM paths are theoretically motivated associations, not demonstrated effects, and some relations are plausibly reciprocal (for example, reliance may both follow from and deepen anthropomorphic attachment). No intervention or behavioral data were collected, so all directional and interventional statements are framed as hypotheses for longitudinal and experimental test; the convergent interview evidence, drawn from a second data source, only partially offsets these single-method, cross-sectional limits. All measures were self-reported, including perceived AI literacy, which by construction captures self-assessed rather than tested competence; future work should pair self-report with objective literacy tasks and behavioral indicators of reliance. Because recruitment relied on an anonymous convenience and peer-referral strategy, we could not control the sample’s composition: it was geographically concentrated in Shandong (96.1%, chiefly Jinan and Qingdao), predominantly female (76.8%), and skewed toward first-year students (60.5%). This regional and cohort imbalance is an inherent feature of anonymous convenience sampling, in which respondents’ locality and year of study cannot be steered; it limits generalization beyond this profile. Future work should sample more deliberately across regions and year cohorts. Three constructs were measured with two items; their reliabilities were generally adequate but the two-item trust scale was marginal in the primary sample (Spearman–Brown = 0.69) and should be lengthened in future work. The specific condition identified by NCA as necessary (social norms) should be read cautiously, both because norms are near-universal in this sample and because necessity results can vary with sample composition. Finally, several conditions were strongly skewed, which destabilized the fsQCA configurational solutions even at *N* = 565; we therefore based inference on SEM and NCA and reported fsQCA only as an exploratory probe.

## 8 Conclusion

Generative AI has, within the span of two years, become a routine cognitive partner for a generation of students—and the stakes are highest among those preparing to teach, whose own habits of mind will shape how their future pupils learn to think with, or lean on, these tools. Against this backdrop, the present study moves the question of GenAI dependency in pre-service teachers beyond documenting that it exists toward explaining why it takes hold and what, if anything, holds it back.

Three findings stand out. Behavioral dependency is already the norm rather than the exception; it is driven less by rational appraisals of usefulness than by dispositional impulsivity and an affective, almost companionable attachment to the tool, with a supportive peer environment serving as a necessary backdrop; and the competence most often prescribed as a remedy—AI literacy—proves only a modest safeguard, its protective value partly offset by the trust and normative immersion that travel with it. Methodologically, by triangulating variable-centered (SEM), necessity (NCA), and configurational analyses, the study shows that net effect and necessity are distinct questions a single method would have conflated. The deeper challenge these results pose is an uncomfortable one: if the students most fluent with AI are not the most protected from depending on it, then teaching people to use AI better may not, by itself, teach them to need it less. Cultivating the self-regulation and self-awareness to set a capable tool down—precisely when it is easiest to keep leaning on it—may prove the more difficult, and more consequential, educational task of the AI era.

## Data Availability

The data will be made available upon reasonable request made to the corresponding author.
