# Supplementary material for: Addicted to AI? Prevalence and psychological drivers of generative AI dependency among pre-service teachers
Source: Front Psychol. 2026 Aug 12;17:1922716. doi: 10.3389/fpsyg.2026.1922716 (PMC13506420; doi:10.3389/fpsyg.2026.1922716)
Supplement: Supplementary file 1 [file Supplementary_file_1.docx]

**Appendix A. The Generative AI Dependency Inventory (GAIDI)**

| **Construct / Item** | **Loading** |
| --- | --- |
| **AI dependency (DEP)*** |  |
| Even for tasks I could do myself, I habitually hand them to GenAI first | .84 |
| I increasingly struggle to complete study/lesson-prep tasks without AI | .83 |
| I adopt GenAI’s answers directly rather than thinking deeply myself | .88 |
| I often can’t be bothered to verify GenAI’s conclusions and just use them | .81 |
| **Impulsivity (IMP)** |  |
| Rather than working through things slowly, I prefer getting an answer immediately | .78 |
| If something takes long effort before it pays off, I easily lose patience | .84 |
| **AI anxiety/FOMO (ANX)** |  |
| The pace of AI development makes me uneasy | .59 |
| I worry over-using GenAI will make me “dumber” | .59 |
| Seeing others better at GenAI makes me feel pressure | .81 |
| I worry I’ll fall behind if I don’t keep up with the latest GenAI tools | .89 |
| Seeing classmates use GenAI for efficiency, I worry about losing out | .90 |
| I fear missing the shortcuts or opportunities GenAI can bring | .81 |
| **Trust in AI (TRU)** |  |
| I believe GenAI’s answers are generally accurate | .67 |
| I am willing to make decisions based on GenAI’s suggestions | .79 |
| **Anthropomorphic attachment (ANTH)** |  |
| Interacting with GenAI feels like talking to a real person | .79 |
| Sometimes I feel a sense of closeness or companionship toward GenAI | .82 |
| **Perceived AI literacy (LIT)** |  |
| I know how to ask GenAI clear, effective questions (prompts) | .79 |
| I can judge whether GenAI’s answers are reliable and need verification | .83 |
| I can select the appropriate GenAI tool or function for different tasks | .84 |
| **Social norms (NORM)** |  |
| Most classmates around me use GenAI to support their learning | .94 |
| Using GenAI is seen as completely normal in my peer community | .86 |

*Note.* * self-designed Behavioral Generative AI Dependency Scale (B-GAIDS)

Administered in Chinese; English translations shown. 7-point Likert. Loadings from the primary-sample CFA (N = 565).

Three items were removed during validation (a reverse-worded impulsivity item, a cross-loading social-norms item, and a weak social-norms availability item); see Section 3.2.

Two items on the AI anxiety/FOMO scale carry standardized loadings below the .70 ideal — Q18 and Q19. Both were reviewed and retained deliberately, on the following grounds: 1) Both load significantly on the intended factor (*p* < .001), and both exceed the conventional .50 acceptability floor for standardized loadings (Hair et al., 2019). 2) Convergent validity holds with them included: the six-item ANX scale returns AVE = .602 (> .50) and CR = .898 (> .70), so the Fornell–Larcker and composite-reliability criteria are both satisfied. The remaining four items (λ = .81–.90) anchor the factor, so the two lower loadings do not depress AVE below threshold. 3) Content validity would be narrowed by removal. The two items capture distinct, theoretically central facets of AI anxiety/FOMO — apprehension about the pace of AI development (a FOMO-type unease) and worry about cognitive deskilling — that the four higher-loading items do not fully represent. Trimming them to raise average loadings would sacrifice construct coverage for a cosmetic gain. 4) The measurement model reported here is the confirmatory model and was pre-specified before the confirmatory analysis; item-level modification to maximize loadings was not undertaken, consistent with retaining a theoretically specified construct rather than a data-driven one.

**Appendix B. Semi-structured Interview Guide**

*Note.* Interviews were conducted in Chinese; the guide is shown here in English translation. Questions were used flexibly, with follow-up probes and requests for concrete examples.

**Opening and background**

- Consent and anonymity confirmation.
- Please introduce your major, year of study, and general learning situation.
- How did you first come to know about AI tools, and what prompted you to start using them?

**Use patterns**

- Which AI tools have you used (dialogue, slides, design, etc.)? Which do you use most, and why?
- What do you mainly use AI for—please distinguish your own learning from teaching/lesson preparation.

**Impulsivity and offloading**

- In what situations do you turn to AI first, and when do you choose to work on your own? Can you recall a concrete example?
- Have you noticed a change in your learning efficiency or outcomes because of AI? Please give an example.
- What is the greatest benefit AI brings to your learning or lesson preparation?

**Anxiety, norms, and environment**

- Do you see using AI as an active choice (it is genuinely useful) or a passive one (having to keep up)?
- Have you ever felt that “everyone is using it, so I will fall behind if I do not”?
- Do you and your classmates exchange AI experience? Have you felt any peer pressure or anxiety around AI use?
- What is your school’s / teachers’ attitude toward your use of AI, and how do you view that attitude?

**Trust and verification**

- Have you encountered AI output that was wrong, outdated, or inaccurate? How do you judge and handle this?
- In lesson design, roughly what proportion of AI-generated content is usable? Which types do you trust more or less?
- Are you concerned that your data, lesson plans, or papers could be collected by the AI platform? Does this affect your use?

**Attachment and human–AI relationship**

- What does an ideal human–AI collaboration look like to you? What role do you want AI to play in your teaching (assistant, partner, tool)?

**Dependency and agency**

- When using AI for assignments or papers, have you ever hesitated over whether it counts as cheating? How did you handle it?
- Do you worry that long-term AI use will erode your independent thinking or lesson-design ability?
- Have you found yourself becoming dependent on AI—times when you cannot start without it?
- Where do you draw the line between AI doing the work for you and AI assisting you?
- Have you ever felt guilt or unease from using AI? Where do you think that feeling comes from?

**Professional identity and the future**

- Over the next 3–5 years, how will AI change how pre-service teachers learn and do their practicum? Could there be a “no AI, no job” situation?
- What about future teachers can AI never replace?
- If AI becomes ever stronger at transmitting knowledge, will the teacher’s role shift away from knowledge transmission?
- Have you prepared for your own irreplaceability—deliberately training abilities AI lacks?
- Has AI affected your confidence in or expectations of becoming a teacher—more excited, or more anxious?
- If a student said, “Teacher, AI explains this better than you—why should I listen to you?”, how would you respond?

**Closing**

- Looking back over the interview, is there anything you would like to add?
- What one suggestion would you give to AI developers or education policymakers about how AI could better serve the preparation of pre-service teachers?
